# Technology-Enabled Intervention to Enhance Mindfulness, Safety, and Health Promotion Among Corrections Professionals: Protocol for a Prospective Quasi-Experimental Trial

**DOI:** 10.2196/45535

**Published:** 2023-09-22

**Authors:** Diane Elliot, Kerry Kuehl, Carol DeFrancesco, Wendy McGinnis, Susanna Ek, Allee Van Horne, Katherine Ginsberg Kempany

**Affiliations:** 1 Health Promotion & Sports Medicine Oregon Health & Science University Portland, OR United States; 2 Oregon Department of Corrections Salem, OR United States

**Keywords:** corrections professionals, mindfulness, total worker health, occupational safety, digital, health promotion, safety, depression, suicide, obesity, cardiovascular disease, well-being, stress, survey, healthy eating, physical activity, mood, vascular health, injury, cop, police, security, undercover, remand, detention, prison, state correctional, state corrections, correction, penitentiary

## Abstract

**Background:**

Correction professionals are a highly stressed workforce with heightened risks for depression, suicide, obesity, cardiovascular disease, and injury. These professionals, largely hidden from view, have received little study concerning means to improve their safety, health, and well-being. In other settings, mindfulness has resulted in lowered stress, along with other benefits. We hypothesized that a program that promoted mindfulness combined with more typical health and safety components could uniquely benefit corrections professionals.

**Objective:**

This project will assess a novel scalable, self-administered program to enhance the mindfulness, safety, and health of a vulnerable worker group.

**Methods:**

In partnership with the Oregon Department of Corrections, we are conducting a prospective quasi-experimental trial of a safety, health, and mindfulness program among 100 corrections professionals from 2 institutions. Survey and physiologic data will be collected at enrollment, upon weekly program completion (3 months), and at 9 months after enrollment. Primary outcome behaviors promoted by the program are being mindful, healthier eating, more physical activity, and greater restorative sleep. Secondary downstream benefits are anticipated in stress level, mood, positive feelings about the organization, vascular health, and cellular aging, along with job performance, injuries, and economic costs. Participants will meet in-person or in a Zoom-type meeting as 3- to 5-member coworker groups during their usual work hours for 30-minute sessions once a week for 12 weeks. The program uses self-guided web-based learning modules that include brief mindfulness practice, and it is accessible by smartphone, tablet, or laptop. Daily mindfulness practice is encouraged between sessions, which is facilitated by the study website and group format. The modules’ structure emphasizes prerequisite knowledge, peer support, skill practice, self-monitoring, and enhancing self-efficacy for change. The program continues through self-directed use of the Headspace app following the 12 weekly sessions.

**Results:**

Participants are being enrolled, and the intervention is ready to launch.

**Conclusions:**

Although mindfulness training has gained traction for worker well-being, its usual format requires a skilled trainer, an initial retreat, and weekly 2-hour meetings for several weeks. The content is limited to mindfulness without safety or health promotion aspects. The need for skilled trainers and time commitment limits the scalability of the usual mindfulness interventions. The planned program is an innovative combination of technology, e-learning, and a group format to add mindfulness to a safety and health curriculum. If acceptable and effective, the format would facilitate its widespread use.

**Trial Registration:**

ClinicalTrials.gov NCT05608889; https://classic.clinicaltrials.gov/ct2/show/NCT05608889

**International Registered Report Identifier (IRRID):**

PRR1-10.2196/45535

## Introduction

### Background and Rationale

Those working in correctional facilities experience high stress levels, which negatively impact their mental and physical health [1-5]. The US Department of Justice published a review that highlighted the many sources of stress in corrections work, such as hypervigilance, constant threats of violence, media scrutiny, a closed work environment, understaffing, organizational issues, and work or family conflicts [1]. These workers have high rates of depression [6], suicidality [7], obesity, and other cardiovascular disease risks [4]. Corrections professionals also experience twice the rates of injury, sick leave, and health care costs than other government employees [8,9]. These professionals, largely hidden from view, have received little study concerning means to improve their safety, health, and well-being.

We are conducting a prospective proof-of-concept acceptability and initial efficacy trial of a scalable, technology-enabled worksite mindfulness, safety, and health promotion program specifically designed for corrections professionals. The unique program adds to our prior worker health and safety programs [10-12] by adding mindfulness. Mindfulness is especially applicable because of its ability to mitigate the effects of stress [13-15]. Our prior National Institutes of Justice-funded work with this population provided a basis for stress mitigation in corrections [16,17]; in that project, we surveyed corrections professionals and measured their perceived stress using the Perceived Stress Scale (PSS-4 Short Form). Using a linear mixed effects regression model, we found that perceived stress increases with increased work-related stress (*P*=.02), work hours (*P*=.03), work-life imbalance (*P*=.002), and lack of procedural justice (*P*=.03) [16]. Those findings helped inform this program’s content. We also stratified participants by their stress level, and the higher and lower stress subgroups completed brain functional magnetic resonance imaging assessment of decision-making under emotionally charged conditions [17]. It is known that decision-making is affected by stress [18], and the ability to make quick, thoughtful decisions particularly is relevant for corrections work. We found that the officers who have higher stress needed significantly more top-down cognitive control, suggesting that their decision-making may be impaired in certain situations. Similar alterations in executive function have been found among young adults with a history of chronic stress [19,20].

Those detrimental effects on cognitive function, as well as other stress-related mental and physical adverse effects, can be mitigated by mindfulness [14]. A 2023 systematic review of mindfulness interventions among law enforcement identified 8 studies demonstrating promising benefits on reducing stress [21]. Several years ago, the Oregon Department of Corrections (DOC) used mindfulness training within a subset of corrections professionals [22]. However, as in most worksite studies, mindfulness training required a major time commitment and skilled trainers [15]. For example, the Oregon DOC program involved 2, day-long workshops plus 2 months of 2 hours per week for its trainer-led sessions. We sought to use a new format for mindfulness training, using a strategy that would be more scalable.

We are adding mindfulness to a program that is designed to promote Total Worker Health (TWH) [23]. The term Total Worker Health, trademarked by the US Centers for Disease Control and Prevention, refers to the combination of policies, programs, and practices that integrate protection from work-related health hazards with the promotion of illness prevention. As the founding members of the National Institute for Occupational Safety & Health-funded Oregon Healthy Workforce Center, we have been at the forefront of TWH efforts and have developed programs that positively impacted first responder groups and home health care workers [10-12].

Mindfulness generally is not a component of TWH programs, and our challenge was to design a scalable TWH program that added mindfulness training. The program is called Next Level, taking the traditional TWH program up a notch with mindfulness. We also recognize that many TWH programs have not been used outside of their study setting [24], and to further maximize dissemination potential, outcome data go beyond participant behaviors and include indices of compensable injuries and work performance [25].

### Objectives

Our overarching aim is to create a scalable and effective program of mindfulness and TWH for corrections professionals. We hypothesized that the intervention would result in beneficial effects related to primary outcomes of dispositional mindfulness, as well as behaviors favoring health promotion and health protection.

### Design

This study will use a quasi-experimental pre-post design to compare values of outcome measures at 3 times points: baseline, immediately post intervention (3 months after baseline), and follow-up (9 months after baseline). Randomization of the intervention by site or individual is not feasible due to personnel movement and contamination effects within and across correctional facilities. An advantage of the pre-post design is that each participant will act as their own control, increasing the power of the study [26]. Each of the 2 participating corrections facilities will be enrolled sequentially and will be a new wave of the project to allow managing activities across the 2 dispersed sites.

The theoretical underpinnings of the intervention are based on our validated model of effective behavior change [27]. In establishing that model, we combined mediation analyses across our prospective randomized behavior change trials with different populations and established that effective programs included prerequisite knowledge, normative beliefs, and self-efficacy for change. Each of those features was built into this program.

Practicing mindfulness will be addressed similarly to other behaviors, such as healthy eating and achieving regular physical activity. However, unlike eating and exercise, mindful meditation will be a new ability for many participants requiring skill acquisition and practice. Accordingly, mindfulness practice is included in each session with a focus on its relationship to that session’s safety or health topic. The program also emphasizes behavioral self-monitoring and peer norms to reinforce Social Cognitive Theory’s tenant that observing progress toward objectives can enhance self-efficacy [28,29].

## Methods

### Setting and Participants

Eligible participants are corrections professionals working on units from the Oregon State Correctional Institution and Coffee Creek Correctional Facility. Corrections professionals are security and nonsecurity staff working for pay at the facility. Enrollment criteria include being employed for at least 6 months, with the exclusion criteria of not planning to leave or retire during the next 9 months. The total number of eligible participants is estimated to be approximately 600, with a budget for 100 participants. Following informational meetings with the administration hierarchy and union officials, we will use existing communication channels to inform potential participants and personally meet with them at their preshift group meetings, when information concerning the prior shift is shared. Individuals will not be directly compensated but will receive release time to participate. Enrollment is on a first-come, first-serve basis, and we expect to reach our 100-participant goal during a 6-month recruitment window. We have used similar strategies successfully in our prior studies of first responders [10,11]. We will carefully track all enrolled participants using the CONSORT (Consolidated Standards of Reporting Trials) guidelines [30].

### Ethics Approval

The Oregon Health & Science University (OHSU) institutional review board (IRB; #24303) has approved the study and its procedures.

### Confidentiality

The OHSU IRB requires an explicit safety and monitoring plan, and it supervises project safety and reporting of any adverse events. All data and all participant responses will be confidential and stored securely. All participants will be assigned a unique, nonmeaningful identifying code number, necessary for longitudinal participant tracking that will correspond to their Headspace participant identification number, to allow merging Headspace website analytics with other data. The study data will be collected and managed using Qualtics and REDCap (Research Electronic Data Capture; Vanderbilt University) electronic data capture tools [31,32]. Qualtrics offline surveys is an application that allows securely administering surveys on a mobile device without an internet connection. REDCap is a secure web-based software platform designed to support data capture for research studies. Both are approved by the OHSU IRB for data collection. Audio files of focus groups will be recorded on OHSU password-protected devices and downloaded to an OHSU password-protected secure site. Hard copies of observation information will be stored in a locked file cabinet at OHSU. No identified individual data will be presented or published. Individual data may be shown without identifying the participant to illustrate scientific principles.

### Intervention

The intervention will be delivered during twelve 30-minute weekly meetings among coworkers. The Oregon DOC supports the project by providing paid time during participants’ usual work days to participate. The sessions are built using Articulate 360, which is a well-established subscription platform for e-Learning course development [33]. It includes access to authoring apps and a library with thousands of course assets. For example, it has hundreds of potential dynamic components, such as interactive games, quizzes, and reveals. It provides a consistent learner experience across all devices (desktop computer, smartphone, or tablet).

**Figure 1:**
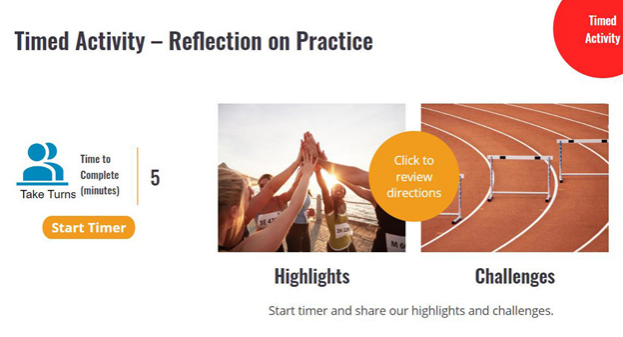
Shared reflection on prior session’s objectives.

**Figure 2:**
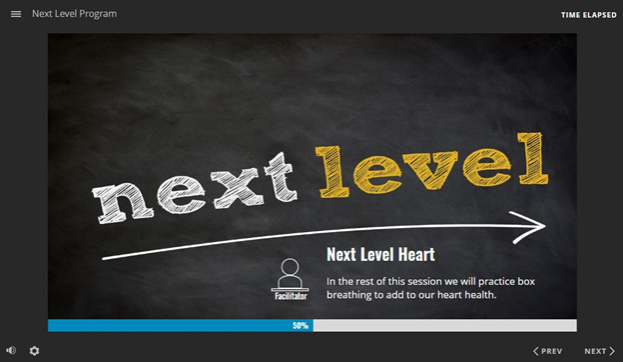
Transition to add mindfulness to traditional Total Worker Health topic.

**Figure 3:**
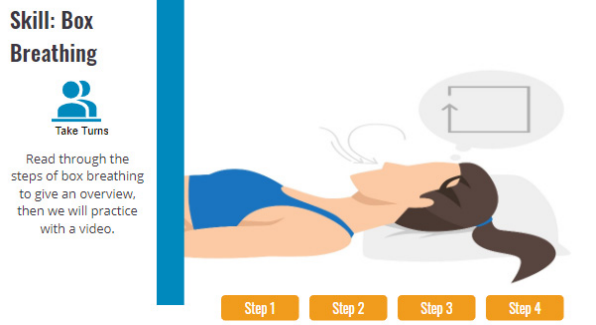
In-session introduction to a skill that is followed by video-led skill practice.

**Figure 4:**
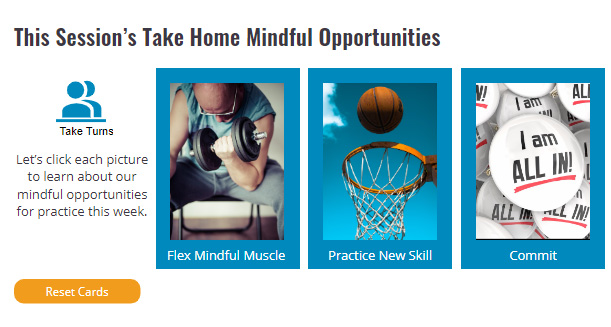
Participants click on each picture and read the opportunities (goals) for mindfulness practices for that week.

Each of the sessions contains approximately 20 wireframes. The wireframes are the baseline structure to which features (eg, games, quizzes, reveals, matching, videos, and links to other content) can be attached. Each module follows a similar format. The sessions are done with 3 to 6 coworkers positioned around a large video screen or sharing a screen when meeting in a Zoom-type meeting. Early in each session, there is a 5-minute follow-up on behavioral objectives (or practice opportunities) from the prior session (Figure 1). This provides a forum to problem-solving challenges, enhance self-efficacy for efforts, and engage in social support. Next, foundational knowledge for that session’s content is presented and actively processed by completing quizzes, reading reveals, or watching a 2-minute video. The session transitions to mindfulness with a “Next Level” wireframe (Figure 2). During the second half of the session, participants either practice a seated meditation or a new mindfulness skill related to that session’s topic. For example, in Healthy Heart, they learn and practice box breathing (Figure 3). The session wrap-up presents daily mindfulness practice objectives for the coming week, which will be revisited in the following session (Figure 4).

Our experience and the literature [34] emphasize that corrections professionals feel that their unique stressors require an authentic understanding of their issues, and accordingly, the content is specifically related to their profession. In addition, we believed that corrections professionals frequently lacked a background or intrinsic interest in mindfulness [35]. Consequently, the initial 2 sessions were designed to build motivation by providing background relevance, a scientific basis for mindfulness, and testimonials from corrections professionals, as well as an overview of the program’s content, process, and how they will run the sessions. These sessions involve a series of videos and voiceover wireframes. Beginning in session 3, on-screen icons guide participants to run the session themselves, with 1 of them as the designated facilitator for that session. The scope and sequence of the twelve, weekly 30-minute sessions are shown in Table 1.

**Table 1:** Next-level scope and sequence.

Session	Content	Mindful in-session practice	Take home daily practice
1	Background and motivation building	Two-minute guided meditation	Daily 2-minute meditation+explore the website
2	Format for and running the sessions	Two-minute guided meditation	Daily 2-minute meditation+explore the website
3	Healthy heart	Box breathing	Daily 3-minute meditation+box breathing twice this week
4	Physical fitness	Three-minute guided meditation	Daily 3-minute meditation+walking meditation twice this week
5	Healthy eating	Mindful eating	Daily 3-minute meditation+mindful eating three times this week
6	Mental fitness	Three minute-gratitude meditation	Daily 3-minute meditation+gratitude meditation at least twice this week
7	Halfway check-in	Three-minute guided meditation	Daily 3-minute meditation+watch introduction Headspace video
8	Sleep	Body scan	Daily 5-minute meditation+bedtime body scan twice this week
9	Injury prevention	Three-minute guided meditation	Daily 5-minute meditation+visit Headspace app
10	Resilience	Three-minute guided meditation	Daily 5-minute meditation+visit Headspace app
11	Medical check-up	Three-minute guided self-compassions meditation	Daily 5-minute meditation+at least 2 self-compassion meditations
12	Wrap-up and next steps for next level	Three-minute guided meditation	Daily 5-minute meditation+visit Headspace app

The program does not require a trained instructor. The coworkers, guided by icons on the screen, are responsible for running the sessions. The initial session has videos and voice-over wireframes, and during session 2, the group-managed format is presented. The group is directed to identify 1 member as the designated facilitator, who follows those icons’ directions. However, being the facilitator requires no preparation or special expertise and other group members can easily assume that role if that individual is not present.

We have used similar coworker-led sessions successfully with other occupational groups [10-12]. We particularly wanted to avoid a hierarchical pedagogic structure of “teaching to” corrections professionals. Peer-led programs have documented efficacy and may be as effective as professionally delivered formats [36,37]. Peers also reduce expenses, promote a sense of shared norms, enhance social support, and augment material with credible examples and personal experiences [38].

To provide a longitudinal program component, we partnered with Headspace, an established effective mindfulness app. In a recent review of mindfulness training apps, Headspace received the highest rating [39]. Individuals will have continuous access to the Headspace app beginning at enrollment for a total of 12 months. Transitioning to more Headspace use is incorporated in the latter weekly sessions to maintain mindfulness once completing the weekly sessions tailored to corrections professionals.

**Figure 5:**
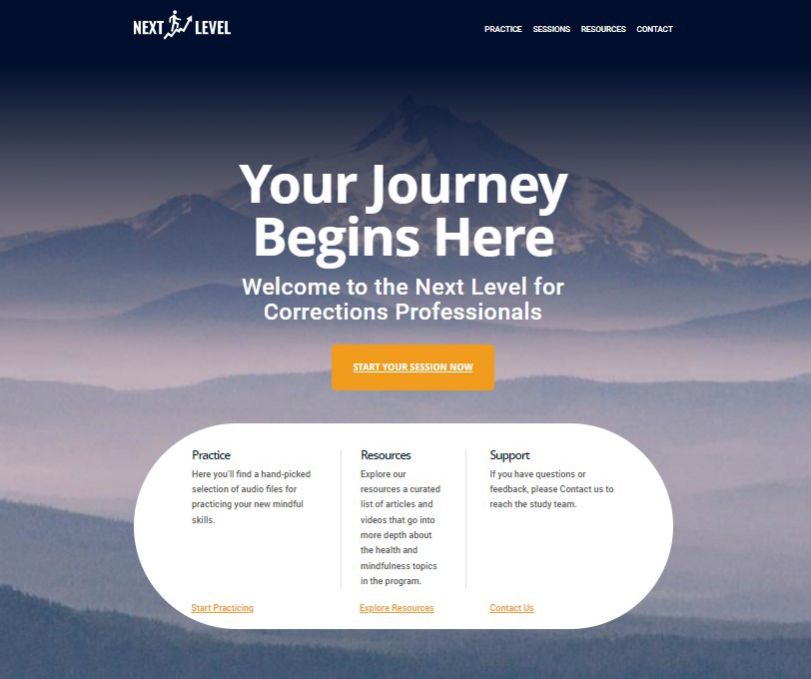
Landing page for the Next Level website.

The program is accessible from a simple website portal designed specifically for corrections professionals. It functions on all operating systems. While corrections professionals often do not have their smartphones with them on duty, they do have access to the meeting and breakrooms with computers, tablets, and internet access. A mockup of that the website landing page is shown in Figure 5. Menu pages include (1) the sessions, (2) audio files of guided mindfulness meditations for use during their daily practice, (3) resources with PDFs and links to videos of interest and related to session topics, and (4) Headspace information. For each session, there is a link to play the session, along with buttons to review the video used in that session, and a restatement of the take-home practice opportunities for that session.

### Outcomes

Three types of outcome data will be collected at baseline, immediately after the 12-week intervention, and at 9 months (6 months following the intervention’s conclusion): (1) Surveys using validated self-report measures for demographic variables, mindfulness indices, current (if any) mindfulness practices, healthy behaviors (nutrition, physical activity, sleep, and safety actions), work-life balance, mental health, perceived stress, and perceptions of the organization or worksite; (2) biomeasures: blood pressure, height, weight, vascular reactivity, and blood work for cellular senescence; and (3) economic outcomes: missed days, overtime, sick days, injuries, incidents, and workmen’s comp claims.

These variables along with citations to the validated instruments are shown in Textbox 1.

**Outcome measures.**

**Primary outcomes**

- Mindfulness (Mindful Attention Awareness Scale [40,41] and Facet Mindfulness Questionnaire [42])
- Self-compassion [43]
- Gratitude [44]
- Eating habits [11]
- Mindful eating [45]
- Physical activity [46]
- Mindful physical activity [47]
- Sleep [48,49]

**Secondary outcomes**

- Perceived stress [16]
- Centers for Disease Control and Prevention health days [50]
- Feelings about the organization [16]
- Mood [51-53]
- Other biometrics (height, weight, and blood pressure)
- Vascular reactivity [54]
- Telomere length [55]
- Health care costs (overtime, days missed, injuries, incidents, and worker compensation claims)

**Other**

- Session observations for fidelity, dose, and reach
- Application metrics
- Follow-up focus groups

The primary outcomes relate to behaviors directly promoted by the program: being mindful; healthier eating; more physical activity; and greater restorative sleep. Secondary downstream benefits are anticipated in perceived stress; negative affect; greater positive feelings about the organization; vascular health or cellular aging; and job performance/injuries/economic costs. All survey constructs have established validity, and many have been used in prior studies. In addition, the baseline survey will include demographic and work history items. The post intervention survey also will include items related to intervention usability, acceptance, enjoyment, and whether they would recommend the program to other corrections professionals.

We will track injury counts, absenteeism (used as a proxy for productivity), worker’s compensation claims, and costs associated with illness and injury. We will collect data on individuals’ reported illness, injury (slips, trips, falls, exposures, and incident interactions) before enrollment and prospectively during and for 2 months following the study. We will use 2-month data for participants, including 2 months prior to enrollment, months 3 and 4 after beginning the program, and a third 2-month interval after completing the study. Absenteeism will be indexed with the Institution Staff Deployment System. We will track pre- and postprogram unusual incident reporting, which is a DOC method of tracking the activity of consequence to the good order, such as inmate self-injury, uses of force, blood and bodily fluid exposure, contraband, and individual or group disturbances.

There are 2 unique biomeasures used in this study: digital thermal monitoring and markers of cellular senescence. Digital thermal monitoring is a cost-effective test of vascular reactivity and endothelial function. It is well correlated with Framingham Risk Score, Coronary Artery Calcium Score, and computerized tomography angiography. It is measured with the Endothelix DTM system, which is approved by the US Food and Drug Administration, noninvasive, and easy to use (VENDYS, Endothelix Inc). With that system, a blood pressure cuff occludes arm arterial flow for 5 minutes. Following cuff deflation, reactive hyperemia is largely dependent on endothelial cells. The healthier the artery, the larger the hyperemic response. This assessment can show changes in response to health promotion interventions and provide a proximal index of positive cardiovascular effects.

We also will assess cellular senescence. This is a measure known to be accelerated by stress and reversed by effective wellness interventions [56-58]. Changes in senescence are associated with a heightened risk for disease and mortality. At present, there is no “gold standard” for cellular senescence. We will use a combination of methods to assess cellular aging: cellular staining for senescence-associated β-gal (Senescence Cells Histochemical Staining Kit, Sigma); the presence of senescence-associated proteins using Western blot; and senescence marker antibody sampling kits to stain for p16, p21, phospho-histone H2AX, Lamin B1, HMGB1, matrix metalloproteinase 3, cytokine interleukin 6, and tumor necrosis factor α. We also will determine the telomerase activity in cell extracts through its ability to synthesize telomeric repeats onto an oligonucleotide substrate, which is amplified by polymerase chain reaction, using the TRAPEZE RT Telomerase Detection kit (Sigma). The relative quantification of gene expression will be determined using the 2^−ΔΔCt^ method with control RNAs appropriate for blood, and a comprehensive senescence phenotype profile will be obtained using the Human Cellular Senescence array (SA Biosciences).

### Fidelity and Adherence

We will observe approximately half of the sessions and use the session’s wireframes as a checklist for fidelity, as well as note taking during session observations concerning participation, engagement, and any deviations from the planned format. When possible, observations will done be in person, and we also will join group Zoom-type meetings. We will assess fidelity (the extent to which the intervention is delivered as intended), dose (how much is delivered), and reach (the proportion of intended recipients participating). A simple email check-in will be a final session component, where participants self-report attendance and indicate session duration. In addition, participants’ visits and time on the Headspace app will be tracked with that app’s analytics.

### Qualitative Data Collection

Following the intervention, we will conduct focus groups concerning program use and outcomes. We will offer partaking in a focus group to all participants. When possible, we will have individuals who went through the sessions together also be together in a focus group. These will last 45 to 60 minutes and will use a trained facilitator and interview guide designed to explore the study outcomes and session observations. The focus group roadmap or interview guide is included in Multimedia Appendix 1. A scribe will take notes and track participant comments.

### Power and Sample Size

We expect a sample size of 100 participants from 2 facilities. We first estimated an approximate dropout rate of 20%, leaving 80 participants. For the 4 primary outcome constructs, we adjusted the α level using Bonferroni corrections by dividing .05 by 4 giving us an α level of .0125 to account for multiple testing. In order to power the change in outcomes from baseline to post intervention (3 months), we used paired *t* tests. We estimate that this sample size has 90% power to detect a moderate effect size. We estimate (Cohen *d*=.41), 85% power to detect (*d*=.43), and 90% power to detect (*d*=.46). All power analyses were conducted using PASS 2019 (NCSS, LLC). For qualitative data, rather than a specified number of groups, data gathering will continue until saturation, when no new information is obtained.

### Data Analysis

Preliminary analyses will include careful examination of descriptive statistics and distributions for variables at each time point. This will include counts and proportions for categorical variables and means, SDs, and range for continuous variables, as well as 95% CIs for all proportions and means.

### Primary Outcomes

Our primary criterion for determining intervention efficacy will be differences in changes in the primary outcomes listed in Textbox 1. Our primary hypothesis is that the intervention will be associated with an improvement in each of those outcomes. Repeated measures linear mixed effects models will be used to determine whether the outcome measures improved from baseline to post intervention. Models will include terms for the time points T_3_ (0 vs 3 months) and T_9_ (0 vs 9 months), and nested random effects for individuals within sites. Primary analyses will test the significance of the terms to determine whether outcome measures change from baseline to post intervention. Models will adjust for age and gender and follow a purposeful selection strategy to determine which other background characteristics to include. Mixed effects models have the advantage that participants do not need complete data for all 3 time points to be included in the analyses. If after data inspection it is decided to dichotomize some measures, logistic mixed effects models will be used instead.

### Secondary Outcomes and Associations

Secondary outcomes will be analyzed similarly using repeated measures mixed effects models with linear or logistic links depending on whether the outcomes are continuous or binary. We will furthermore analyze whether the changes in primary outcomes are associated with changes in other selected measures. In particular, we hypothesize that (1) reduced stress and healthier behaviors (eg, nutrition, physical activity, and sleep) are associated with improvements in vascular reactivity and cellular senescence and (2) reduced stress and improved perceptions of the organization are associated with reduced injuries and incidents.

### Qualitative Outcomes

We are experienced in mixed methods, having used them successfully in understanding program outcomes [48,59,60]. Analyses of the focus groups following the intervention will involve reiterative pattern analysis of comments, which avoids the substantive bias inherent in a priori categorization. Instead, we will adapt the more inductive constant-comparative method typical of grounded theory [61]. This analysis approach increases the transparency and reproducibility of qualitative research. Our aim is to further explore the findings observed in the primary and secondary outcomes, beginning with a roadmap of questions and exploring the responses. Barriers and enhancements to program impact will be sought within the dynamic contexts of actual experience. Construction of themes and interpretations rather than bounding interpretation with a prespecified list of categories for coding avoids the substantive bias inherent in a priori categorization. Instead, we will use an adaption of the more inductive constant-comparative method [62,63]. Following the identification of themes, the data set will be subjected to microreview in a search for both confirming and disconfirming data. Quotations will be selected to exemplify and elaborate themes, support findings, and illuminate contexts, promoting reader understanding and facilitating. We will use applicable items from the COREQ (Consolidated Criteria for Reporting Qualitative Research) checklist to document and communicate our methods [64]. In addition to providing a deeper understanding of outcomes, the qualitative findings should allow the development of case studies that may be useful in sharing findings with the corrections community.

## Results

As of December 2022, this study is enrolling participants and finalizing the web-based curriculum and website. We anticipate beginning the intervention in early 2023, with data collection to be completed by November of 2023.

## Discussion

Fifty years ago, Kabat-Zinn et al [65] used Mindfulness-based Stress Reduction to lessen stress and distress for those with chronic pain [65]. MBSR takes time and generally includes a day-long retreat, weekly 2.5-hour classes for 2 months, and daily activities including around 40 minutes of meditation. Although its effectiveness is well established [66], its time commitment is a hurdle*.* Work-related stress is estimated to result in losses of up to US $300 billion annually in the United States [67]. Mindfulness training has gained some traction in the worksite setting [68,69] but still has limited use, partially due to the need for a significant time commitment and an experienced trainer. The innovative format described in this protocol overcomes 3 challenges: time, tailoring, and TWH incorporation. It is delivered in a 30-minute once-a-week, self-directed format and designed specifically for corrections professionals. Importantly, it is incorporated in a TWH context. Mindfulness and TWH are rarely combined. A literature search for TWH and mindfulness yielded only a single citation. Some evidence suggest that mindfulness training may be more effective when combined with other components [70].

Our outcome findings will include three main dimensions: (1) implementation acceptability, observations for fidelity, and adherence; (2) pre- to postsurvey measures of targeted behaviors and in particular dimensions of mindfulness along with measures of inflammation and cellular senescence, in addition to human resource data on missed work, injury claims, and incident reports; and (3) postparticipation survey items relating to program acceptability and focus group interviews concerning for a deeper understanding of practical issues, social validity, and suggestion to improve the program and its use.

The 3 listed dimensions reflect 3 components of this protocol. The first dimension relates to whether we crafted a program that was easy to use and engaging enough to sustain participation, especially in the context of the stressful and generally understaffed context of corrections. The second dimension relates to changes in behaviors with an emphasis on instruments used to assess mindfulness following the usual longer form of mindfulness interventions. The focus group aspect of the third dimension will allow a more thorough understanding of the program, as well as provide suggestions for improvement.

We recognize that each correctional system has its unique structural, organizational, and personnel factors and that our findings may not generalize to corrections professionals in other settings. In addition, this is the first evaluation of the peer-led, technology-enabled weekly session and self-directed practice format to increase mindfulness, hence the emphasis on observing sessions and formative qualitative feedback in debriefing after the trial. In addition, participants are not a random sample of these workers. However, we have site-wide data from our previous work [16], and we will be able to compare participants with other corrections professionals from these sites. Despite limitations, the innovative aspects of the program will provide outcome numbers that could inform a subsequent larger randomized controlled trial of the program in this and other settings.

## Appendix

Multimedia Appendix 1 Focus group interview guide.

## Abbreviations

CONSORT: Consolidated Standards of Reporting Trials
COREQ: Consolidated Criteria for Reporting Qualitative Research
DOC: Department of Corrections
IRB: institutional review board
PSS-4: Perceived Stress Scale
OHSU: Oregon Health & Science University
REDCap: Research Electronic Data Capture
TWH: Total Worker Health

## Data Availability

The data sets generated and analyzed during this study will be available in the National Archive of Criminal Justice Data repository.
