# Supplementary material for: Technology-Enabled Intervention to Enhance Mindfulness, Safety, and Health Promotion Among Corrections Professionals: Protocol for a Prospective Quasi-Experimental Trial
Source: JMIR Res Protoc. 2023 Sep 22;12:e45535. doi: 10.2196/45535 (PMC10559194; doi:10.2196/45535)
Supplement: Multimedia Appendix 1 [file resprot_v12i1e45535_app1.pdf]

IRB Approved: 10/6/2022
Approval Expires: 10/5/2023

OREGON HEALTH & SCIENCE UNIVERSITY

IRB # 24303

Focus Group Script

Before beginning the interview OHSU researcher states:

- *Thank you for your time. The goal today is to talk for about 45 minutes and get your feedback on the total worker health and mindfulness program you completed.*
- *Participation is* ***voluntary****.*
- *Please speak freely; there are no right or wrong answers. You do not need to feel that you are speaking for anyone other than yourself. We are interested in each individual's viewpoint.*
- *Since this is a research study, we will take notes. Notes will be kept confidential with OHSU research staff. These responses will not be shared with your managers, coworkers, or anyone who is not part of the OHSU research staff.*
- *Information gathered with be grouped from other interviews, no individual names will be used in group summaries. OHSU researchers will review the information gathered and identify patterns that emerge. With this information, we will modify the learning modules.*
- *Please refrain from using your last name or other full names during this interview to help maintain confidentiality*

Your input today will help shape this program with the goal of improving the health and safety of corrections professionals throughout the country.

We will now begin the interview, we have consented all who are participating and will begin.

1. To start, what do you remember most from completing some of the program?
2. What did you enjoy?
3. What sessions were most helpful and why?
4. Did you go through the modules individually or in a group or both?
    a. Describe the setting?
    b. What format worked best
5. What advice would you give other COs running the program?
6. What health habits did you change? (sleep, washing, smoke exposure, nutrition etc)
7. What else would you like to tell us about the program?
8. What is your advice on dissemination methods to COs?
9. You were given access to the Headspace app; what did you find useful?
10. What helped you remember to practice mindfulness?

THANK YOU –

IRB Approved: 10/6/2022
Approval Expires: 10/5/2023

This concludes the interview. We appreciate your participation. Do you have any questions? If you have any additional questions, please contact us.

Interview Notes

Type of interview (circle one)**:** **Individual / Focus Group**

Interviewer Name: __________________________

Date: _______ / ______ / _______

Time: Start _______ : ______ AM / PM

End _______ : ______ AM / PM

Total _________ minutes

Location (select one):

Station:
